# Recent advancement in prevention against hepatotoxicity, molecular mechanisms, and bioavailability of gallic acid, a natural phenolic compound: challenges and perspectives

**DOI:** 10.3389/fphar.2025.1549526

**Published:** 2025-03-21

**Authors:** Peng Chen, Fanzhao Zou, Wei Liu

**Affiliations:** ^1^ Department of Pharmacy, Renmin Hospital of Wuhan University, Wuhan, Hubei, China; ^2^ Renmin Hospital of Wuhan University, Wuhan, Hubei, China; ^3^ Department of Geriatrics, Renmin Hospital of Wuhan University, Wuhan, Hubei, China

**Keywords:** hepatotoxicity, DILI, gallic acid, molecular mechanisms, bioavailability

## Abstract

Drug-induced liver injury (DILI) results from the liver toxicity caused by drugs or their metabolites. Gallic acid (GA) is a naturally occurring secondary metabolite found in many fruits, plants, and nuts. Recently, GA has drawn increasing attention due to its potent pharmacological properties, particularly its anti-inflammatory and antioxidant capabilities. To the best of our knowledge, this is the first review to focus on the pharmacological properties of GA and related molecular activation mechanisms regarding protection against hepatotoxicity. We also provide a thorough explanation of the physicochemical properties, fruit sources, toxicity, and pharmacokinetics of GA after reviewing a substantial number of studies. Pharmacokinetic studies have shown that GA is quickly absorbed and eliminated when taken orally, which restricts its use in development. However, the bioavailability of GA can be increased by optimizing its structure or changing its form of administration. Notably, according to toxicology studies conducted on a range of animals and clinical trials, GA rarely exhibits toxicity or side effects. The antioxidation mechanisms mainly involved Nrf2, while anti-inflammatory mechanisms involved MAPKs and NF-κB signaling pathways. Owing to its marked pharmacological properties, GA is a prospective candidate for the management of diverse xenobiotic-induced hepatotoxicity. We also discuss the applications of cutting-edge technologies (nano-delivery systems, network pharmacology, and liver organoids) in DILI. In addition to guiding future research and development of GA as a medicine, this study offers a theoretical foundation for its clinical application.

## 1 Introduction

The liver accounts for approximately 2% and 3% of the body weight in adults and adolescents, respectively, and is the largest internal parenchymatous organ of the body (Juza and Pauli, 2014). This organ performs physiological functions by regulating glycolipid and protein metabolism, stimulating the secretion of bile, and detoxifying products, such as drugs, plasma ammonia, and ethanol (Reinke and Asher, 2016; Driskill and Pan, 2021; Almazroo et al., 2017). Thus, the liver is highly vulnerable to toxins. Drug-induced liver injury (DILI) refers to the liver injury caused by adverse drug reaction, including chemicals, biological products, traditional Chinese patent medicines, health products, dietary supplements, as well as their metabolites, excipients, or impurities, which can deteriorate from asymptomatic liver dysfunction to liver failure and even death (Andrade et al., 2019; Li X. et al., 2022). DILI is typically divided into intrinsic and idiosyncratic categories based on the potential mechanism of drug action (Hoofnagle and Bjornsson, 2019). Intrinsic DILI is dose-dependent and predictable, with a relationship between the cytotoxic properties of the causative drug, whereas idiosyncratic DILI is primarily host-dependent and unpredictable because of its interactions with environmental and host elements (Andrade et al., 2019; Chen et al., 2015). The annual incidence of DILI in China is 23.80 per 100,000, and the two leading causes of DILI were traditional Chinese medicines and anti- -tuberculosis (TB) drugs (Shen et al., 2019).

Oxidative stress (OS) refers to a state of imbalance between oxidant agents and antioxidant effects in the body, with a tendency towards oxidation, which is recognized as one of the most central mechanisms participating in the progression of liver diseases, especially DILI (Cichoz-Lach and Michalak, 2014; Villanueva-Paz et al., 2021). In addition to OS, a number of other processes are involved in DILI, including activation of the immune response (Liu et al., 2004; Andrade et al., 2023), bile salt export pump inhibition (Morgan et al., 2010) and direct damage to toxicological drug properties (Price et al., 2009). Here, we discuss the role of OS in DILI. OS leads to generation of reactive radical species (ROS), included by superoxide radical (O_2_·-), nitric oxide (NO·), hydroxyl radical (HO·), peroxynitrite (ONOO·), and hydrogen peroxide (H_2_O_2_), which exert an influence on mitochondrial dysfunction and endoplasmic reticulum stress (Zorov et al., 2014; Uzi et al., 2013). Therefore, an acceptable therapeutic strategy using natural antioxidants instead of conventional treatments may ameliorate liver injury. Simultaneously, many plants and their extracts have been added to liver injury therapy owing to their antioxidant properties.

Gallic acid (GA) is a natural bioactive phenolic compound with antioxidant, anti-inflammatory, antimutagenic, anticarcinogenic, antiviral, antiultraviolet, and antimicrobial properties, contributing to the protection of organs against toxic compound-induced injury (Fanaei et al., 2021; Shruthi and Shenoy, 2021; Wang et al., 2023a; Govea-Salas et al., 2016). Despite the numerous pharmacological actions of GA, its function in DILI has not been elucidated. Thus, this paper aimed to provide a more conceptual and novel insight by reviewing the scientific evidence related to the hepatoprotective effects of GA, indicating that GA has the potential to be extensively used in medical treatments to attenuate liver injury caused by toxicity.

## 2 The chemistry and source of GA

GA monohydrate appears as white or yellowish needle-shaped crystals or powder and was discovered in 1786 b y Scheele. The molecular weight of GA is 170.12 g/mol (Akbari et al., 2019), with a relative density of 1.694, and it is also a relatively thermostable compound (melting point of 252°C and boiling point of 501.1°C). It is soluble in ethanol and ether; hardly soluble in cold water and methanol; and insoluble in benzene and chloroform. Heating to 100°C–120°C will result in the loss of crystal water from GA. Further heating above 200°C will lead to the loss of carbon dioxide and formation of pyrogallic acid. The molecular formula of GA is C_7_H_6_O_5_; structurally, it contains one carboxyl group and three hydroxyl groups attached at positions 3, 4, and 5 on a benzene ring.

**FIGURE 1:**
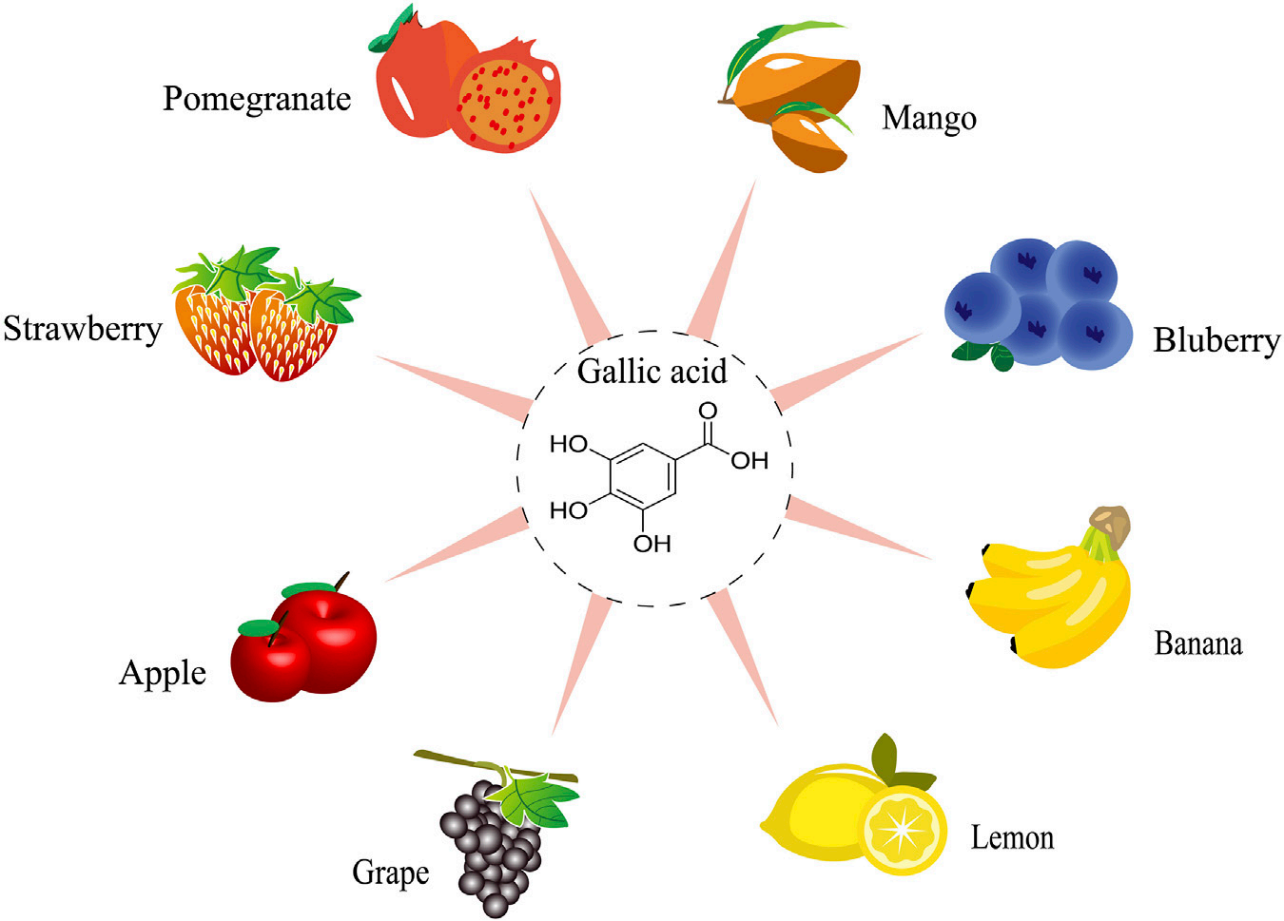
The resources of gallic acid from fruits.

GA is a dietary polyphenol found in various fruits, vegetables, plants, and nuts, such as pomegranates and grapes, *Acacia confuse* Merr. and *Graptopetalum paraguayense* E. Walther (Tung et al., 2009; Duh et al., 2011; Zhou et al., 2019). Here, we discuss the sources of GA in various fruits (Figure 1). According to the Duke’s Phytochemical and Ethnobotanical Databases, mangoes have the highest average GA concentration, reaching up to 9,000 mg per 100 g of fresh fruit. Up to 95% of all polyphenols have been found in mango pulp from several commercial types and these polyphenols are primarily GA and galloyl-derived polyphenols, such as mono-galloyl glucose and gallotannins (Kim et al., 2021). Furthermore, pomegranates are thought to contain the second highest concentration of GA in any fruit, ranging from 0.45523 to 2045.00000 mg/100 g (Rothwell et al., 2013). Some fruits with lower GA content (such as strawberries, bananas, lemons, and apples) promote gastric acid secretion and encourage gastrointestinal peristalsis due to their natural phenolic acid content, which is beneficial for the digestion and absorption of food in the body. In addition, GA is present in a wide range of plants and vegetables, including *Mentha spicata* L., *Camellia japonica* L., *Guazuma ulmifolia* Lam., and *Momordica charantia* L. As anticipated, it has been determined that the majority of fruits and plants have the ability to eliminate heat, detoxify, and induce diuresis, all of which help to partially explain the biological actions of GA.

## 3 The toxicity of GA

GA combines critical proteins or minerals, such as zinc, calcium, and iron to form an insoluble complex that disturbs other bioactive substances. *In vivo* studies have been performed with oral administration of GA to animals, and the edible LD_50_ value for GA is 5,000 mg/kg in rabbits and >2000 mg/kg in mice (Variya et al., 2019; Niho et al., 2001). However, limited information is available regarding the long-term toxicity of GA. Hematological studies showed no discrepancies in serum biomarkers (ALT, AST, GGT, and ACP) (Abarikwu et al., 2016). Histopathological findings demonstrated that only a small number of adipocytes were inhibited without bone marrow suppression. In addition, Variya et al. verified that a high dose of 900 mg/kg GA administered orally to Swiss albino mice daily for 28 days showed no significant morphological and behavioral alterations, and the histopathological findings proved the safety of GA (Variya et al., 2019).

Fischer rats were orally administered 0%–5% GA for 13 weeks. The toxic effects following 5% and 0.6% in females and males, respectively, included anemia (reduction of red blood cell counts, hematocrit, and hemoglobin concentration, and an increase in reticulocytes) and liver cell hypertrophy (Niho et al., 2001). Based on the present toxicology data *in vivo* models, 0.2% GA is regarded as a no-observed-adverse-effect level, which translates to 119 and 128 mg/kg/day for male and female rats, respectively (Niho et al., 2001). GA exhibits anti-tumor properties by inducing apoptosis in cancerous cell lines; however, it can also be harmful to normal cells by causing chemical changes in the GA molecule (Park et al., 2007; You and Park, 2011; Park et al., 2008). The major difference in cytotoxic potential between molecules is the length of the carbon chain, which influences the physical and chemical properties of GA (including its solubility and dispersion coefficient). Furthermore, changes in the propagation potential through the lipid membrane can affect interactions between molecules and their intracellular targets. The lipophilicity of GA determines its pharmacological activity and drug reaction, which leads to its binding to other targets that induce DNA damage (Locatelli et al., 2008).

## 4 Antioxidant properties of GA

In most cases, liver damage involves oxidative stress, which gradually evolves into hepatitis, cirrhosis, and hepatocellular carcinoma (HCC) without proper therapy. GA contains three adjacent reductive phenolic hydroxyl groups in its structural formula, which can bind to surrounding free radicals. Therefore, the antioxidant properties of GA play a considerable role in alleviating the accumulation of free radicals and delay the progression of liver damage (Duh et al., 2011; Gholamine et al., 2021; Wu et al., 2023). A 25-μM dose of GA exerts antioxidative effects by decreasing the inflammatory mediators (such as TNF-α, IL-1β, MCP-1, and iNOS) and promoting the expression of antioxidant enzymes in a co-culture of lipid-laden HEPA one to six hepatocytes and RAW 264 macrophages (Tanaka et al., 2020). GA and its derivatives can neutralize hypochlorous acid (HOCl) to protect α-1-protease against the inactivation, and reduce the peroxidation of the phospholipids in brain when dissolved in ethanol (Aruoma, 1993). In animal models of DILI, the activity of serum antioxidant biomarkers (including glutathione [GSH], superoxide dismutase [SOD], catalase [CAT], and GPx) significantly increased and lipid peroxidation products decreased in the GA-treated group compared with those in the model group (Go et al., 2016; Omobowale et al., 2018; Esmaeilzadeh et al., 2020). Altogether, GA exhibits antioxidative effects on liver damage as well as on other diseases associated with oxidative stress, such as cancer, cardiovascular disease, degenerative disease, and metabolic disease (Bashar et al., 2021; Heidarian et al., 2016; Yan et al., 2019; Mori et al., 2020). Therefore, GA is a potential dietary supplement owing to its ability to scavenge ROS and improve antioxidant capacity.

## 5 Anti-inflammatory properties of GA

Liver diseases, such as HCC, are linked to underlying chronic inflammation and fibrosis caused by drug/environment toxicity, alcoholic and non-alcoholic fatty liver diseases, and hepatitis virus infections (Marengo et al., 2016; Yang et al., 2019). The anti-inflammatory properties of GA have been extensively investigated in recent *in vivo* and *in vitro* studies. In an *in vitro* experiment, GA inhibited secretion of pro-inflammatory cytokines (TNF-α, IL-1β, and IL-6), thereby preventing the damage response of lipopolysaccharide (LPS)-stimulated murine BV-2 cells (Lin et al., 2015). GA can impede intracellular receptor-interacting protein (RIP)1 and RIP3 increases and extracellular high mobility group box 1 protein (HMGB1) elevation induced by ethanol in an *in vitro* study of LO2 cells, resulting in inhibition of hepatocyte necroptosis (Zhou et al., 2019). Consistently, GA significantly protected against alcohol-induced stomach ulcers by lowering submucosal edema and cell infiltration in a dose-dependent manner by blocking the production of TNF-α and IL-1β/6 (Zhou et al., 2020). The downregulation of these pro-inflammatory factors by GA may be due to action of some transcriptional factors and kinases, such as c-JUN N-terminal kinases (JNK), NF-κB, and mitogen-activated protein kinase (MAPK) (Fanaei et al., 2021; Lin et al., 2015; Cai et al., 2024). Taken together, these results demonstrate that GA has anti-inflammatory properties and can serve as a powerful anti-inflammatory agent.

## 6 Anti-hepatotoxic properties of GA

Hepatotoxicity is defined as liver dysfunction or damage caused by exposure to drugs or xenobiotics during liver metabolism (Anita and Om, 2014). Jaundice, edema, and discomfort are the first signs of liver disease, and these symptoms are accompanied by biochemical alterations, including elevated blood levels of hepatic enzymes and reduced hepatic enzymatic activity (Anita and Om, 2014). The examination of histology often reveals the common findings of destruction of intracellular organelles, fatty degeneration and necrosis of central hepatocytes, along with fibrosis and cirrhosis (Omobowale et al., 2018; Chen et al., 2018; Safaei et al., 2018). Hepatotoxicity could be caused by the combined action of the primary substance and reactive metabolites, as well as by immunologically mediated reactions that affect hepatocytes, biliary epithelial cells, the liver vasculature system, and other organs or tissues (Deng et al., 2009). Reactive metabolites refer to the metabolic pathways activated by drugs (including free radicals, quinones, unstable conjugates, and epoxides) that can combine with macromolecular substances (such as proteins and nucleic acids) intracellularly and interfere with the normal metabolism or structure of cells (Attia, 2010). Collectively, GA showed hepatoprotective properties against various liver-damaging agents in rodent models (Table 1).

**TABLE 1:** The hepatoprotective effects of gallic acid under various conditions of liver damage models in vivo.

No.	Condition	Animal	Modeling dose and time	Treatment	Effects	Signaling pathway	Findings	References
1	Carbon tetrachloride -induced liver damage and inflammation	Male Sprague Dawley rats 4-week-old	CCl_4_ solution (CCl_4_/olive oil = 1/4) 0.5 mL/kg by oral administration; twice a week for 8weeks	WGP 50 and 300 mg/kg by oral administration Daily for 8 weeks	AST↓; ALT↓; MDA↓; GSH↑; TNF-α↓; TC↓; TG↓; GPx↑; CAT↑; SOD↑; GR↑; TBARs↓		Scavenge free radical and suppress serum enzymes representing liver toxicity	Duh et al. (2011)
2	Carbon tetrachloride-induced hepatotoxicity	Male Institute Cancer for Research mice; 10-week-old	CCl_4_ solution (CCl_4_/olive oil = 1/9) 0.7 mL/kg A single dose by IP injection	GEGR 250, 500 and 1,000 mg/kg distilled water by intragastric gavage Daily for 5 days	ALP↓; AST↓; ALT↓; SOD↑; MDA↓; TNF-α↓; IL-6↓; IL-10↑; TGF-β1↓	p-P38↑; p-JNK↓; MMP-2↓; MMP-1↑; Smad2/3↓	Suppress lipid peroxidation by increasing the expression of antioxidant enzyme	Go et al. (2016)
3	Carbon tetrachloride-induced liver fibrosis	Male BALB/c mice Weighing 18–22 g	CCl_4_ solution (CCl_4_/olive oil = 3/7) 3 mL/kg by SC injection Once a week for 8 weeks	GA 5 and 15 mg/kg by intragastric gavage Daily for 6 weeks	HA↓; cIV↓; MDA↓; ALT↓; AST↓; γ-GT↓	MMP-2↓; TIMP-1↓	Alleviate liver fibrosis and improve liver function	Wang et al. (2014)
4	Carbon tetrachloride-induced acute liver injury	Male Wistar rats 8-week-old and weighing 180–200 g	Exposed to CCl_4_ vapor for 20 min	GA 240 mg/kg by oral administration; daily for 3 days GA 0.24 mg/kg by IP injection; daily for 3 days	GOT↓; GPT↓; O_2_ radical-scavenging activities in serum↑; O_2_ radical-scavenging activities in hepatocyte↓		Prevent the progression of liver injury by stabilizing cell membranes	Kanai and Okano (1998)
5	Carbon tetrachloride-induced chronic liver injury	Male Sprague Dawley rats Weighing 220–330 g	CCl_4_ solution (CCl_4_/olive oil = 2/3) 0.75 mL/kg by SC injection Once a week for 6 weeks	ACBE 50, 250 mg/kg by oral administration; daily for 8 weeks GA 50 mg/kg by oral administration; daily for 8 weeks	AST↓; ALT↓; CAT↑; GRD↑; GPx↑; MDA↓; CYP2E1↓; TBARs↓; GSH/GSSG↑; TG↓; TC↓		Increase antioxidant enzyme expression and inhibit CYP2E1 activation	Tung et al. (2009)
6	Carbon tetrachloride- induced acute and chronic hepatotoxicity	Male Wistar albino rats Weighing 180–200 g	CCl_4_ solution (CCl_4_/olive oil = 1/1) 1 mL/kg by single IP injection CCl_4_ solution (CCl_4_/olive oil = 3/7) 3 mL/kg by IP injection twice a week	GA 100 mL/kg by oral administration; daily for 7 days GA 50 and 100 mg/kg by oral administration; twice a week for 4 weeks	ALT↓; AST↓; GGT↓; TBARS↓; GSH↑; GPx↑	p53↑	Improve nonenzymatic antioxidant level and increase the expression of p53 gene	Perazzoli et al. (2017)
7	Doxorubicin-induced hepatotoxicity	Male Wistar rats Weighing 120–180 g	Dox 15 mg/kg A single dose by IP injection	GA 60 and 120 mg/kg by oral administration Daily for 7 days	ALT↓; ALP↓; T-Bilirubin↓; MDA↓; SOD↑; CAT↓; H_2_O_2_↓; GSH↑; GPx↑; NO↑; GST↑; NPT↑; TT↓		Decrease oxidative markers by strengthening antioxidant defense system	Omobowale et al. (2018)
8	Methotrexate-induced toxicity	Male Wistar rats Weighing 180–220 g	MTX 20 mg/kg A Single dose by IP injection	GA 30 mg/kg by oral administration Daily for 10 days	AST↓; ALT↓; MDA↓; ALP↓; GSH↑; SOD↑; GPx↑; CAT↑		Enhance the decreased antioxidant enzymes activity to improve antioxidant defenses	Safaei et al. (2018)
9	Cyclophosphamide-induced toxicity	Swiss albino mice 8–10-week-old and weighing 23–27 g 3 females and 2 males per group	CP dissolved in distilled water 50 mg/kg A single dose by IP injection	GA dissolved in 0.5% carboxymethyl cellulose 100, 200 and 400 mg/kg by oral administration Daily for 5 days	SOD↑; GSH↑		Improve gene-protected properties and elevate biomarkers of antioxidant defense system	Shruthi and Shenoy (2021)
10	Cisplatin-induced nephrotoxicity and hepatotoxicity	Male Wistar albino rats 8–10-week-old and weighing 150–250 g	Cisplatin 3 cc per rat for once on the day 4 in a 7-day study	GA and silymarin dissolved in water 8 mg/kg by gastric gavage	CAT↑; MDA↓; GSH↑; 8OH-dG↓; SOD↑; Urea↓; Creatinine↓		Ameliorate oxidative stress through increasing antioxidant enzymes	Dogan et al. (2022)
11	Diclofenac-induced liver toxicity	Male Wistar rats 6-8-week-old and weighing 180–220 g	DIC 50 mg/kg by IP injection Daily for 5 days	GA 50 and 100 mg/kg by oral administration Daily for 5 days	AST↓; ALT↓; ALP↓; T-Bilirubin↓; MDA↓; GSH↑; SOD↑; GPx↑; CAT↑; IL-1β↓; ferric reducing/antioxidant power↑		Suppress inflammatory response and improve antioxidant enzymes	Esmaeilzadeh et al. (2020)
12	Acetaminophen-induced acute liver injury	Male Swiss albino mice Weighing 25–35 g	APAP dissolved in saline 400 mg/kg A single dose by IP injection	E.G., dissolved in distilled water 10 and 20 mg/kg A single dose by oral administration	AST↓; ALT↓; LPO↓; SOD↑; CAT↑; GSH↑; GST↑; GR↑; GPx↑; TNF-α↓; IL-1↓	NF-κb↓; p65↓; p52↓; COX2↓	Prevent the decrease of antioxidant and repress inflammatory response	Ezhilarasan et al. (2024)
13	Paracetamol-induced liver damage	Male crossbreed Swiss albino mice Weighing 20–25 g	Paracetamol 900 mg/kg A single dose by IP injection	GA dissolved in saline 100 mg/kg A single dose by IP injection	ALT↓; AST↓; ALP↓; GPx↑; GR↑; GST↑; lipid peroxidation↓; TNF-α↓; SOD↑; CAT↑		Mitigate the accumulation of inflammatory mediators and reverse the depletion of antioxidants	Rasool et al. (2010)
14	Ethanol-induced acute intoxication	Adult female Sprague Dawley albino rats Weighing 180–220 g	Ethanol 8 mL/kg A single dose by oral gavage	GA dissolved in saline solution 50, 100 and 200 mg/kg A single dose by oral administration	ALT↓; AST↓; LDH↓	paraoxanse (PON)↑; arylesterase↑	Rejuvenate the antioxidant enzymes activity and activate PPARγ to ameliorate liver damage	Kartkaya et al. (2013)
15	Ethanol-induced liver disease	Male C57BL/6J mice 8-week-old and weighing 16–20 g	52% alcohol solution 7.5 mL/kg by oral gavage Daily for 12 weeks	SL-GAC 100, 200 and 400 mg/kg by oral administration Daily for 12 weeks	ALT↓; AST↓; γ-GT↓; serum and liver iron↓; UIBC↑; TS↓; MDA↓; SOD↑; TC↓; TG↓; GSH↑	TfR1↓; Hepcidin↑	Suppress the downregulation of hepcidin and adjust the expression of TfR1 to ameliorate hepatic damage	Wu et al. (2019)
16	Fluoxetine-induced oxidative stress and liver damage	Male Wistar rats 10–12-week-old and weighing 180–220 g	Fluoxetine dissolved in pure corn oil 24 mg/kg by oral administration Daily for 4 weeks	GA solution (GA/ethanol-distilled water = 1/10) 50, 100 and 200 mg/kg by oral administration Daily for 4 weeks	GOT↓; GPT↓; MDA↓; TNF-α↓; CAT↑; SOD↑; Vit C↑; ferric reducing ability of plasma (FRAP)↑; TC↓; TG↓; LDL-C↓; VLDL-C↓; HDL↑; serum protein carbonyl↓		Elevate properties of free radical scavenging to retard progression of liver damage	Karimi-Khouzani et al. (2017)
17	Ketamine-induced oxidative damage	Male Wistar rats Weighing 150–200 g	KET 50 mg/kg A single dose by IM injection	GA 13.5 mg/kg by oral gavage Once a day for 3 days	Protein carbonyl↓; NPSH↓		Alleviate liver damage by improving antioxidant properties	Schimites et al. (2020)
18	Lipopolysaccharide-induced inflammation	Male Sprague Dawley rats Weighing 300–400 g	LPS 50 μg/kg A single dose by IP injection	GJ 10 or 30 mg/kg and GA 1 or 10 mg/kg A single dose by intragastric gavage	AST↓; ALT↓	JNK2/1↓; p38↓	Improve anti-inflammatory properties by suppressing JNK and p38 MAPKs activity	Lin et al. (2015)
19	Aflatoxin B_1_-induced oxidative and inflammatory stress damage	Adult Wistar rats 10-week-old and weighing 181–191 g	AFB_1_ 75 μg/kg by oral administration Daily for 28 days	GA 20 and 40 mg/kg by oral administration Daily for 28 days	AST↓; ALT↓; ALP↓; LDH↓; RONS↓; LPO↓; MPO↓; IL-1β↓; IL-10↑; TNF-α↓; caspase-3↓; SOD↑; CAT↑; GPx↑; GST↑; GSH↑; NO↓		Ameliorate liver damage by regulating the detoxification and abating oxidative stress	Owumi et al. (2020a)
20	Isoniazid and rifampicin-induced liver injury	Male Wistar rats 4-6-month-old and weighing 200–250 g	Isoniazid 150 mg/kg and rifampicin 150 mg/kg by intragastric gavage Once a day for 28 days	GA 50, 100 and 150 mg/kg by oral administration Daily for 28 days	Total oxidant capacity ↓; AST↓; ALT↓; ALP↓; Bilirubin↓; HMGB-1↓; IFN-γ↓; SOD↑; CAT↑; GPx↑; GSH↑	Nrf2-p↑; GCLC↑; PRDX6↑; NF-κB↓; TLR4↓; NOS2↓; IL-1β↓	Promote activation of Nrf2 to increase the expression of antioxidant and inhibit NF-κB activation against inflammatory responses	Sanjay et al. (2021)
21	Dimethylnitrosamine-induced liver fibrosis	Male Sprague Dawley rats; weighing 180–200 g	DMN 3 mg/kg by IP injection; three times in the first week DMN 7 mg/kg by IP injection; three times for 3 weeks	GA 25, 50 and 100 mg/kg by intragastric gavage Daily for 5 weeks	AST↓; ALT↓; ALP↓; TB↓; SOD↑; CAT↑; GSH↑; MDA↓; TGF-β1↓; EGF↓; hydroxyproline↓	α-SMA↓; TIMP-1↓; TIMP-2↓; p-Smad2↓; p-Smad3↓	Improve antioxidant capacity and block phosphor-isoform signaling	Chen et al. (2018)
22	Dimethylnitrosamine-induced acute liver injury	Adult Kunming mice Weighing 18–22 g	DMN 30 mg/kg A single dose by IP injection	GA dissolved in 0.5% carboxymethylcellulose sodium 50 and 100 mg/kg by oral administration Twice daily for 3 days	MDA↓; GSH↑; ALT↓; AST↓; SOD↑; GST↑	HO-1↑; Nrf2↑; GST-α3↑	Mitigate acute liver injury by promoting the induction of HO-1 and GST-α3 via Nrf2 pathway	Ma (2014)
22	Sodium arsenite-induced renal and hepatic toxicity	Male Wistar albino rats; Weighing 200–250 g	SA 10 mg/kg by oral administration Daily for 14 days	GA 10 and 30 mg/kg by oral administration Daily for 21 days	AST↓; ALT↓; ALP↓; MDA↓; NO↓; GSH↑; SOD↑; CAT↑; GPx↑; IL-1β↓; BUN↓; Cr↓		Improve antioxidant capacity by facilitating to scavenge reactive free radicals	Gholamine et al. (2021)
23	Paraquat-induced liver toxicity	Male Wistar rats 6-8-week-old and weighing 180–220 g	Paraquat 25 mg/kg by oral gavage Daily for 14 days	GA 25, 50 and 100 mg/kg by oral administration Daily for 14 days	AST↓; ALT↓; ALP↓; TG↓; TC↓; MDA↓; T-Bilirubin↓; IL-1β↓; SOD↑; CAT↑; LDL-C↓; VLDL-C↓; HDL-C↑; FRAP↑; Vitamin C↑; PC↓		Diminish serum lipid level and oxidative effects caused by paraquat	Nouri et al. (2021)
24	Manganese-induced inflammatory and oxidative sress	Adult male Wistar rats	Mn 15 mg/kg by oral gavage Daily for 14 days	GA 15 and 30 mg/kg by oral gavage Daily for 14 days	ASL↓; ALT↓; ALP↓; LDH↓; GGT↓; RONS↓; LPO↓; MPO↓; NO↓; TNF-α↓; IL-1β↓; SOD↑; CAT↑; GST↑; GSH↑; GPx↑		Exert hepatoprotective effect by limiting oxidative and inflammatory responses	Owumi et al. (2020b)

### 6.1 Carbon tetrachloride (CCl_4_)

CCl_4_ is frequently employed as a model chemical for liver injury to evaluate the effects of hepatoprotective treatments and illustrate the mechanisms underlying hepatotoxic reactions. The unstable free radicals trichloromethyl (CCl_3_·) and trichloromethyl peroxyl (CCl_3_O_2_·), which are produced when cytochrome P450 (CYP450) enzymes metabolize CCl_4_ in the endoplasmic reticulum of hepatocytes (Andritoiu et al., 2014), stimulate Kupffer cells to produce ROS, which results in lipid peroxidation, causing centrilobular hepatic necrosis, inflammation, and fibrosis (Weber et al., 2003). GA ameliorates CCl_4_-induced chronic liver injury by inhibiting lipid peroxidation and suppressing the activity of CYP2E1, a core component of the CYP450 enzyme superfamily and one of the key enzymes in the human body that metabolizes drugs (Tung et al., 2009). According to Perazzoli et al., GA elevates *p53* gene expression in correlation with hepatic GSH concentration, which appears to be related to hepatocyte regeneration and antioxidative responses (Perazzoli et al., 2017). Additionally, GA directly produced antioxidant effects by stabilizing cell membranes and scavenging free radicals (Duh et al., 2011; Kanai and Okano, 1998). Natural extracts containing GA have antifibrotic characteristics, as evidenced by the high expression of metalloproteinase-1 (MMP-1) and the downregulation of profibrotic factors (such as transforming growth factor-β1 [TGF-β1], Smad2/3, matrix metalloproteinase-2 [MMP-2], and tissue inhibitor of matrix metalloproteinase [TIMP-1]) (Go et al., 2016; Wang et al., 2014). -

### 6.2 Antineoplastic drugs

Currently, more than 80 commonly used antineoplastic drugs are roughly divided into six categories (cytotoxic drugs, hormone drugs, biological response modifiers, monoclonal antibody drugs, adjuvant drugs, and other types of drugs) in clinical practice, which are specifically used for cancer therapy. Nevertheless, DILI, a common organ ailment among the adverse responses to antineoplastic medication, limits the therapeutic efficacy of these treatments to some extent. Doxorubicin, an anthracycline class of compounds, has been widely used over the past several decades as a chemotherapeutic drug to treat various types of cancers (including lymphomas, leukemias, breast carcinoma, ovarian carcinoma, and thyroid carcinomas) by altering DNA and producing free radicals, which induce diverse cardiac, hepatic, hematological, and testicular toxicities (Omobowale et al., 2018; Prasanna et al., 2020). In doxorubicin-induced hepatotoxicity, GA enhanced hepatocyte survival by scavenging ROS and bolstering the antioxidant defense system (Omobowale et al., 2018). Cisplatin, cisplatinum, or cis-diamminedichloroplatinum (II) is commonly employed as an anti-tumor agent for treating solid tumors and hematological malignancies, and simultaneously leads to hepatotoxicity, nephrotoxicity, and ototoxicity (Volarevic et al., 2019; Wang et al., 2023b; Dogan et al., 2022). The protective effects of GA were linked to a decrease in oxidative stress and increase in antioxidant enzymes, both of which successfully reduced cisplatin-induced hepatotoxicity and nephrotoxicity caused by cisplatin (Dogan et al., 2022). Cyclophosphamide (CP), one of the most successful antineoplastic agents, shows acute or long-term toxic consequences, including hematological, cardiac, gonadal, hepatotoxic, and other toxic effects (Emadi et al., 2009). GA has anticlastogenic and antigenotoxic effects against CP-induced chromosomal damage in mouse bone marrow cells and reduces the pathological changes in hepatocytes caused by CP, including lobular necrosis, congestion with sinusoid dilatation, irregular arrangement, and rupture of hepatocytes (Shruthi and Shenoy, 2021). In addition, GA can mitigate biochemical and oxidative stress parameters (the reduction of ALT, ALP, malondialdehyde [MDA], and H_2_O_2_; and elevation of GSH, SOD, and CAT) in the liver of Wistar rats exposed to methotrexate, which inhibits dihydrofolate reductase from converting dihydrofolate to tetrahydrofolate to prevent DNA synthesis and cell proliferation (Safaei et al., 2018).

### 6.3 Nonsteroidal anti-inflammatory drugs (NSAIDs)

Owing to their widespread use and efficacy in reducing pain and swelling, NSAIDs are listed on the World Health Organization Model List of Essential Medicines. However, several placebo-controlled trials and meta-analyses have documented the negative effects of NSAIDs on hepatic, renal, gastrointestinal, cardiovascular, cerebral, and pulmonary problems (Bindu et al., 2020). Acetaminophen (paracetamol), originally known as 4-hydroxyacetanilide, is frequently used because of its analgesic and antipyretic qualities. Acetaminophen is the most common cause of drug-induced acute liver failure (ALF) in the United States, with an incidence of 0.59 per 1,000,000 person-years of ALF arising from idiosyncratic DILI (Andrade et al., 2019). Acetaminophen overdose causes a multitude of interrelated metabolic events in hepatocytes, including lipid peroxidation, protein oxidation, covalent modification, oxidative stress, mitochondrial dysfunction, and centrilobular necrosis (Jaeschke et al., 2012). Apart from guarding against oxidative stress and minimizing the damage caused by acetaminophen to the liver, GA also exhibits promise in reducing inflammation by dramatically downregulating pro-inflammatory factors (TNF-α, IL-1, p65, and p52) and upregulating IκB expression in liver tissue, which functions as a blocker for the NF-κB pathway (Rasool et al., 2010; Ezhilarasan et al., 2024). Among NSAIDs with anti-inflammatory, antipyretic, and antinociceptive properties, diclofenac (DIC) has severe adverse effects, including gastrointestinal injury and damage to hepatic, renal, lung, and cardiac tissues (Tomic et al., 2008). GA mitigates DIC-induced liver damage by reducing cellular ROS production, suppressing IL-1β gene expression, replenishing enzymatic and non-enzymatic antioxidants, and improving liver function enzymes (Esmaeilzadeh et al., 2020).

### 6.4 Alcohol

Excessive alcohol consumption poses a substantial public health challenge worldwide. Alcohol can affect all the organ systems in the body, leading to various disorders, most prominently in the liver, where it can cause steatosis, steatohepatitis, cirrhosis, and HCC (Mackowiak et al., 2024). GA stimulated the expression levels of peroxisome proliferator-activated receptor γ (PPAR γ), which is crucial for lipogenesis and lipid production and highly expressed in white adipose tissue (Christofides et al., 2021; Kartkaya et al., 2013). Reportedly, GA reversed the ethanol-induced decrease in paraoxonase (PON) activity by promoting PON1 expression and release from hepatocytes via the PPAR γ-PKA-cAMP intracellular signaling cascade (Kartkaya et al., 2013; Khateeb et al., 2010). In a mouse model of alcoholic liver disease (ALD) with iron overload, Wu et al. found that GA not only boosted antioxidant enzyme activity but also cured ALD-associated iron overload by regulating hepcidin downregulation and decreasing iron absorption in the gut (Wu et al., 2019).

### 6.5 Biotoxins

Biotoxins, commonly known as natural toxins, are chemical substances produced by animals, plants, and microorganisms that exert toxic effects on other biological species. LPS, composed of lipids and polysaccharides, and the primary element of the outer membrane of gram-negative bacteria, results in a significant increase in the release of pro-inflammatory cytokines and ROS, which leads to liver damage (Lai et al., 2023). LPS-induced injury is characterized by the increased expression of nitric oxide synthase (iNOS), IL‐1, IL‐6, TLR4, cyclooxygenase‐2 (COX-2), and prostaglandin E2 (PGE2) (Guo et al., 2021). GA treatment demonstrated antioxidant and anti-inflammatory effects in Sprague-Dawley rats with LPS-induced liver injury by lowering COX-2 expression and JNK2/1 MAPK phosphorylation (Lin et al., 2015). Aflatoxins, as the most studied largely among the mycotoxins produced by fungi, have multiple derivatives, such as aflatoxin B_1_ (AFB_1_), aflatoxin B_2_ (AFB_2_), aflatoxin G_1_ (AFG_1_), and aflatoxin G_2_ (AFG_2_). and AFB_1_ is the most toxic and can cause a variety of health problems, including cancers of the liver, lung, and gastrointestinal tract, delayed development, immunosuppression, and genotoxic effects (Deng et al., 2018; Marchese et al., 2018). Additionally, GA reduces AFB_1_-induced hepatorenal impairment by reversing the increase in caspase-3 levels, inhibiting AFB_1_ activation, and scavenging AFB_1_-O (exo-8,9-epoxide) (Owumi S. et al., 2020).

### 6.6 Antidepressant

Polypharmacy in patients with depression has increased the incidence of antidepressant-induced DILI over the past two decades. Therefore, the hepatotoxicity of various antidepressants has gained interest. Fluoxetine is an antidepressant belonging to the serotonin reuptake inhibitor class and is used as a first-line treatment for depression. It has been proven to cause oxidative damage, which is associated with changes in the liver tissue and blood markers (Inkielewicz-Stępniak, 2011). In addition to reducing oxidative stress and mitigating fluoxetine-induced liver damage, GA modifies the activity of hepatic enzymes that metabolize drugs, such as the inhibition of monooxygenases involved in CYP450 (Karimi-Khouzani et al., 2017). In contrast, GA reduces hyperlipidemia caused by the effects of fluoxetine on the expression of genes linked to fatty acid synthesis and acetyl-CoA carboxylase 1, a lipogenic isoform found in the liver and adipose tissue (Karimi-Khouzani et al., 2017; Cheng et al., 2007). Ketamine (KET) is an anesthetic commonly used in both humans and animals. Recently, it has been shown to effectively and quickly reduce depressive symptoms in individuals with treatment-resistant depression as an N-methyl-D-aspartate receptor (NMDAR) antagonist (Smith-Apeldoorn et al., 2022). Even with acute administration and sub-anesthetic dosage, KET has possible pro-oxidant activity and causes oxidative stress by raising lipoperoxidation while lowering GSH levels, as was previously observed in the prefrontal cortex (De Oliveira et al., 2009). GA administration can prevent and reverse the oxidative damage caused by acute KET administration in brain regions (such as the cortex and hippocampus) and liver, minimizing its noxious effects (Schimites et al., 2020).

### 6.7 Isoniazid and rifampicin

Isoniazid (INH) and rifampicin (RFP) are the first-line drugs used to treat tuberculosis. DILI caused by anti-tuberculosis drugs is the most common in India, accounting for 58% of all cases of DILI and 5%–22% of cases of acute liver failure caused by drugs (Devarbhavi et al., 2010; Kumar et al., 2010). By blocking the main bile salt exporter pump, the basolateral Na^+^/taurocholate cotransporting polypeptide (NTCP), rifampicin causes conjugated hyperbilirubinemia (Mita et al., 2006; Saukkonen et al., 2006). In addition, isoniazid undergoes either direct or indirect metabolism to acetyl hydrazine and hydrazine via N-acetyltransferase (NAT) and amidohydrolase. CYP450 monooxygenases in the liver oxidize these metabolites, producing electrophilic intermediates and free radicals that have been identified as hepatotoxins responsible for liver injury (Tasduq et al., 2005). GA can prevent liver toxicity caused by INH and RFP by maintaining the plasma membrane integrity of hepatocytes, as evidenced by biochemical and histological markers (Sanjay et al., 2021). Moreover, GA has a preventive effect on INH- and RFP-induced liver damage by inhibiting pro-inflammatory signals mediated by NF-κB and upregulating gene production of endogenous antioxidants through the nuclear factor erythroid 2-related factor 2 (Nrf2) pathway (Sanjay et al., 2021). The Nrf2 transcription factor is a major regulator of the antioxidant defense system, which modulates the expression of cytoprotective genes in their regulatory regions and encodes detoxifying enzymes for drug metabolism and redox balance (Kumar et al., 2014). Previous research investigated the inhibitory effects of GA on NF-κB mediated inflammatory response; Kim et al. found that GA reduced NF-κB activation and downregulated the expression of pro-inflammatory cytokines, such as TNF-α and IL-6 (Kim et al., 2006).

### 6.8 Others

Paraquat is an abrasive chemical compound that is widely used to kill plants, particularly for controlling weeds and grasses in developing countries. A blood content of 8.5 μg/mL of paraquat causes harmful effects on organs including the heart, liver, kidneys, and lungs (Amin et al., 2021). The findings of a previous study provided further insight into the free radical scavenging properties of GA by showing that GA (100 mg/kg, po) reduced the levels of plasma protein C, thereby inhibiting the oxidative effects of paraquat on the hepatocytes of Wistar rats (Nouri et al., 2021). Ma et al. demonstrated that GA was able to reduce dimethylnitrosamine (DMN; a potent hepatotoxin, carcinogen, and mutagen)-induced acute liver damage in mice, suggesting the potential mechanism is that detoxifying capacity of liver tissue can be improved by upregulating the expression levels of hemeoxygenase-1 (HO-1) and glutathione-s-transferase-α3 (GST-α3) (Ma, 2014). Simultaneously, another study offered the first evidence that GA, through its enhanced antioxidant capacity and involvement in the regulation of cytokine expression, can reduce DMN-induced liver fibrosis in rats (Chen et al., 2018). Due to its antioxidant qualities, which raise intracellular antioxidant capacity, GA prevented sodium arsenite-induced renal and kidney damage, which was demonstrated by ameliorating tissue histological alterations and serum levels (ALT, AST, ALP, Cr, BUN, MDA, IL-1β, SOD, and CAT) (Gholamine et al., 2021). Additionally, combining polyphenols, such as GA with stable ω-3 fatty acids (ω-3FA) may provide therapeutic drug candidates for treating injuries to target organs caused by oxidative and inflammatory reactions in individuals who have been exposed to manganese or related toxic chemicals at work or in the environment (Owumi SE. et al., 2020).

**FIGURE 2:**
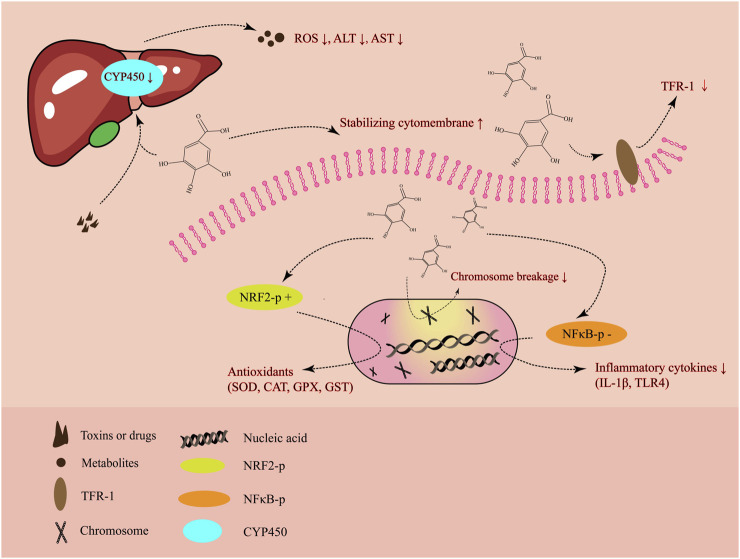
The primary mode of action of gallic acid in alleviating liver damage.

Altogether, through a number of regulatory mechanisms, the most significant of which are its anti-inflammatory and antioxidant properties, GA ameliorates liver damage caused by hepatotoxicity (Figure 2). Without appropriate treatment, liver injury typically progresses to hepatitis, cirrhosis, and even the most serious form, HCC. HCC arising from the hepatocytes, as a late consequence of chronic progressive liver disease, is a malignant tumor that has grown to be a major global public health concern (Severi et al., 2010; Maida et al., 2014). Notably, previous research demonstrated that the Fuzheng Jiedu Xiaoji formulation of traditional Chinese medicine, which contains GA and chlorogenic acid, considerably increased overall and progression-free survival while lowering the death rate of patients with HCC (Yang et al., 2021). Jagan et al. demonstrated that GA is a strong anti-proliferative agent against diethylnitrosamine-induced HCC by lowering the levels of proliferation markers (Jagan et al., 2008). Consequently, GA plays a critical role in the prevention of HCC by ameliorating liver injury and exerting its anti-tumor property.

## 7 Absorption, distribution, metabolism, and bioavailability of GA

The significant biological and pharmacological activities of GA have led to its widespread use in pharmacokinetic testing to study its absorption, distribution, metabolism, and excretion. Evaluation of the optimal dose of GA for disease prevention and treatment is highly beneficial because of these findings.

### 7.1 Absorption of GA

The release of GA from unprocessed dietary ingredients is its primary source of bioactivity. The gastrointestinal tract quickly absorbs dietary GA when administered orally. The absorption of GA in various intestinal segments revealed that the upper end of the gut was superior to the lower end and the absorption process was passive diffusion, based on a rat intestinal unidirectional perfusion model. Furthermore, intestinal efflux transporters, such as P-glycoprotein (P-gp) and multidrug resistance protein 2 (MRP2) may bind to GA (Cheng et al., 2021). Yu et al. conducted comprehensive analyses that identified the following pharmacokinetic properties of GA in SD rats: the maximum plasma concentration (C_max_), terminal elimination half-life (T_1/2_), mean time to peak concentration (T_max_), and area under the curve (AUC) of plasma-concentration time at 0.83 μg/mL, 1.5 h, 2.56 h, and 0.137 mg⋅min/mL, respectively (Yu et al., 2018). Similarly, when rats were orally administered pomegranate flower extract (23.94 mg/kg of GA), T_max_ of 0.5 h, C_max_ of 707.58 ng/mL, and AUC of 5,711.06 (ng⋅h/mL) of GA were noted (Yisimayili et al., 2022). GA and its derivatives are widely found in plants and have a basic chemical structure, therefore, the ability of the gastrointestinal tract to absorb GA following oral administration is dependent on several variables, including sex, age, health state, drug form, and dosage. Therefore, to establish a solid scientific foundation for the dietary supplementation of GA, particular emphasis should be placed on the bioavailability and gastrointestinal absorption mechanism of GA in plant extracts.

### 7.2 Distribution of GA

GA is rapidly absorbed into the circulatory system and distributed to various organs in a wide range of areas. Healthy Sprague-Dawley rats were orally administered *Polygonum chinense* Linn extract equivalent to 2.68 mg/kg of GA. The tissue-to-plasma concentration ratios of GA decreased in the following order: kidneys (6.50), heart (0.99), liver (0.93), lungs (0.473), and spleen (0.155) (Chen et al., 2020). Furthermore, the accumulation of GA in the brain within the first 15 s of the experiment was almost 11 times higher than the basal concentration in the control group, indicating that GA may have brain-targeting characteristics (Gasperotti et al., 2015). As previously indicated, the brain distribution propensity of GA correlates with its protective benefits against neurological disorders caused by medicines, diseases, and environmental toxins; hence, its significance in brain disorders warrants further investigation. The findings of the aforementioned studies indicate that following oral administration, GA is preferentially distributed to the kidney, heart, liver, and brain, and associated with the metabolic processes of the kidney and liver, as well as the pumping function of the heart. Nevertheless, current research has ignored the distribution patterns of GA metabolites in organs, focusing on the detection of these metabolites (such as 3-dihydroshikimic acid, syringic acid, ethyl gallate, and propyl gallate) in plasma, urine, and feces instead (Choubey et al., 2015; Ow and Stupans, 2003). Hence, designing and conducting additional correlation studies between the distribution of GA and its metabolites in tissues, and their biological activities are crucial.

### 7.3 Metabolism of GA

There is a growing consensus that two major phases are involved in the metabolism of GA, and more than 30 metabolites (mostly pyrogallol, protocatechuic acid, methylates, sulfates, and glucuronides associated with these two compounds) have been found in the plasma, urine, and feces (Liang et al., 2013; Zhang et al., 2017). In the phase I metabolism of GA, the principal phase biotransformation pathways are decarboxylation and dehydroxylation processes according to the structure of GA, which produce pyrogallol and protocatechuic acid, respectively, the two main GA-metabolite structures in investigations involving humans (Liang et al., 2013; Zhong et al., 2016). The metabolites of GA are created by sulfation (pyrogallol-2-O-sulfate, protocatechuic acid-3-O-sulfate, and GA sulfate), glucuronidation (GA glucuronide, pyrogallol-O-glucuronidation, and protocatechuic acid glucoside), and methylation (4-O-methylgallic acid) following the creation of phase I metabolites, which are excreted through urine and feces (Wang et al., 2019). The liver is responsible for phase II metabolism of GA, which involves the action of sulfotransferases (SULTs), UDP-glucosyltransferases (UGTs), and catechol-O-methyltransferases (COMTs) (Isvoran et al., 2022; Jarrar and Lee, 2021; Bastos et al., 2017). Similarly, the intestinal microbiota was capable of regulating GA metabolism involving five metabolic reactions (including methylation, dimethylation, trimethylation, decarboxylation, and dehydroxylation) in a cultivation system in which GA and protocatechuic acid were incubated with the intestinal flora fluid of SD rats (Luo et al., 2017).

### 7.4 Bioavailability of GA

Bioavailability is the relative quantity and rate at which drugs are absorbed into the circulation by the body and is influenced by formulation factors (including the size and crystal shape of the drug particles, molecular structure, excipients, tightness of fillers, and manufacturing procedures) and physiological factors (including digestibility, intestinal flora, transporter proteins, and metabolizing enzyme availability) (Kreider et al., 2022). Although GA has been shown to be effective in treating a range of diseases, one of its disadvantages is that it has a low bioavailability due to rapid elimination and poor absorption, which restricts its clinical application and promotion (Shahrzad et al., 2001).

The bioactivity of GA depends largely on its bioavailability, therefore, numerous attempts have been made to increase its bioavailability to enhance its nutritional benefits. In a study using a rat model of hepatotoxicity generated by CCl_4_, a phospholipid complex of GA was designed to improve lipophilicity and prevent poor absorption, boosting antioxidant capacity with enhanced bioavailability (Bhattacharyya et al., 2013). Further investigations found that GA was more stable and sustained when conjugated with phosphatidylcholine or polyamidoamine dendrimers, which enhanced its bioavailability and increased its hepatoprotective effects (Abdou and Masoud, 2018). Furthermore, compared with the polyherbal extracts of amla and pomegranate fruit peels, Patil et al. demonstrated a significant improvement in the oral bioavailability and anti-colon cancer activity of polyherbal nanoparticles (GA isolated from amla and quercetin separated from pomegranate fruit peel extract) (Patil and Killedar, 2021). The incorporation of GA into nanocarriers has the potential to improve oral bioavailability by enhancing its stability in the gastrointestinal tract, increasing its solubility, promoting organ targeting, facilitating barrier penetration, and extending circulation time (Sahyon et al., 2023).

## 8 Future perspectives

**FIGURE 3:**
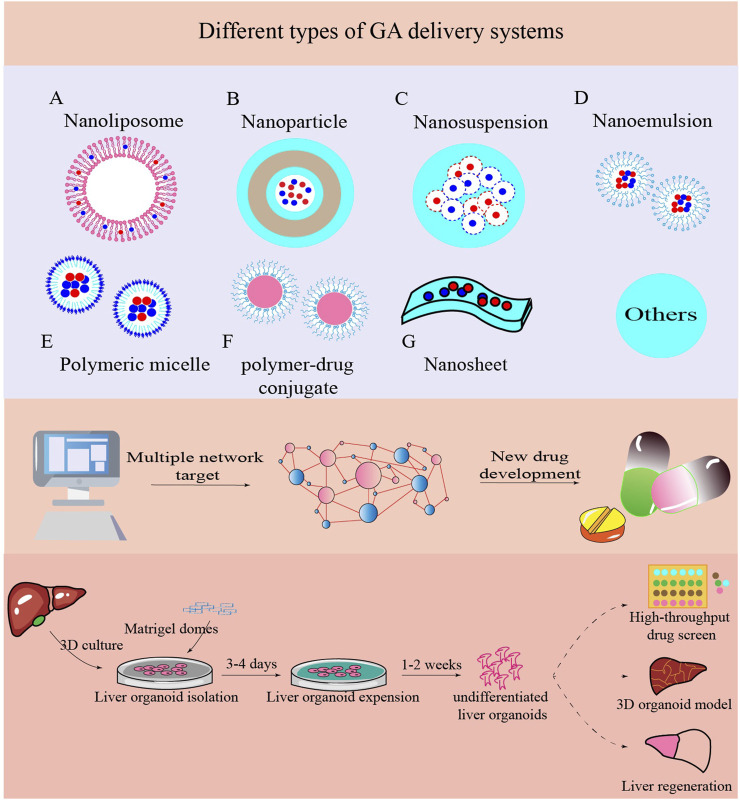
New technologies are applied in liver injury caused by toxins. Nano-delivery systems, network pharmacology, and liver organoid can all be used to reveal more molecular processes in diseases and develop novel treatment strategies.

Due to the low bioavailability of GA, its therapeutic applicability in hepatoprotection is currently limited. Therefore, it is critical to advance our understanding of drug delivery technologies and conduct further preclinical research using *in vivo* and *in vitro* models. The physicochemical properties of small molecules play a major role in determining how well drugs are absorbed into the body, therefore, efforts to improve the solubility, release control, activity range, and pharmacokinetics of drugs should come first in the delivery process. In the relatively new but rapidly expanding fields of nanomedicine and nano-delivery systems, nanoscale materials (liposomes, nanoparticles, polymeric micelles, polymer-drug conjugates, nanosuspensions, and nano-emulsions) are used as diagnostic tools or to carefully distribute therapeutic medicines to precise target areas (Li B. et al., 2022). Simultaneously, it is challenging to attain effective therapeutic results focused exclusively on a single target for a variety of chronic disorders (such as chronic liver damage) caused by multiple factors related to clinical practice. Consequently, network pharmacology has been widely used to discover drugs and active compounds in traditional Chinese medicine. This clarifies the overall mechanism of action, analyzes drug combinations, and determines the formula compatibility to design multi-target molecular drugs, and provide new ideas for the study of complex systems in traditional Chinese medicine and new technological support for rational clinical drug use (Jiashuo et al., 2022; Li and Zhang, 2013; Nogales et al., 2022). Molecular docking, as a chemical calculation method, can theoretically simulate interactions between small molecule medicines and their targets (such as protein receptors), predicting the manner and affinity of binding (Paggi et al., 2024). Combining network pharmacology and molecular docking can significantly increase drug discovery efficiency and contribute to comprehensive evaluation of multi-target mechanisms of action (Zhou et al., 2024; Tan et al., 2023). Moreover, organoid models are more similar to physiological cells in composition and activity, have a more stable genome, and require fewer operations during model development than typical 2D cell lines and animal models. They offer several considerable benefits in cost, clinical relevance, and high-throughput screening (Han et al., 2024; Heydari et al., 2021). Liver organoids can also be used in toxicological research, medication screening, and regenerative medicine, particularly for irreversible liver diseases (Prior et al., 2019; Messina et al., 2020). These new techniques will undoubtedly reveal hitherto unidentified molecular pathways and initiate an important new avenue of research on liver damage caused by hepatic toxicity (Figure 3).

## 9 Concluding remarks

Recently, the scientific community has gradually become interested in GA, a naturally occurring polyphenolic molecule, owing to its antioxidant, anti-inflammatory, and hepatotoxic properties. This paper reviewed recent developments in GA research on its physicochemical properties, fruit origin, toxicity, prevention of hepatotoxicity, molecular mechanisms, pharmacokinetics, and bioavailability. *In vivo* models have provided convincing evidence that GA ameliorated liver toxicity induced by CCl_4_, antineoplastic drugs, NSAIDs, alcohols, biotoxins, antidepressants, anti-tuberculosis drugs, and other environmental toxins. GA is anticipated to be a successful medication for the long-term prevention of DILI because of its weak toxicity in animal and clinical trials. The main mechanisms by which GA reduces DILI include modulation of CYP450 enzyme activity, regulation of the MAPKs signaling pathway, free radical scavenging, upregulation of Nrf2, and downregulation of NF-κB and pro-inflammatory cytokines. Notably, further research is needed on the interactions between GA and other medicines to determine whether GA intervenes in the efficacy of other medicines. Although there is still much to learn about the hepatoprotective properties of GA, preclinical research and clinical trials are required to assess the pharmacological potential of GA metabolites, either in conjunction with medical interventions or as a dietary supplement to prevent toxicity-related liver damage.

Low bioavailability is a characteristic of GA that is influenced by several factors, including absorption, metabolism, distribution, excretion, and disease condition. Consequently, although GA improves DILI, its therapeutic application may be limited due to these disadvantages. Additionally, it is now an accepted practice to enhance the bioavailability of GA and adjust its physicochemical properties using nanotechnology to improve common dosage forms. Common types of nanocarriers include nanoliposomes, nanoparticles, nanosuspensions, nano-emulsions, polymeric micelles, polymer-drug conjugates, and nanosheets. However, more thorough investigations are needed to determine the biocompatibility and stability of the antioxidant activity of these nano-combinations at the animal level. Finally, the quick advancement of cutting-edge methods, such as the use of network pharmacology and emergence of organoids present previously unknown opportunities to research molecular pathways and subsequent pharmacological development in DILI therapy. These groundbreaking investigations could represent a substantial advancement in the search for naturally occurring substances with positive effects on humans and provide a foundation for future research aimed at preventing, mitigating, or curing liver damage caused by xenobiotic exposure or illness. In conclusion, several disorders linked to DILI can be treated with the robust anti-hepatotoxic properties of GA.
